# Intravoxel incoherent motion-based habitat imaging for the prediction of immunohistochemistry in patients with breast cancer

**DOI:** 10.3389/fonc.2025.1595157

**Published:** 2025-06-27

**Authors:** Ailing Wang, Muzhen He, Chengxiu Zhang, Yunyan Zheng, Yang Song, Chenglong Wang, Mingping Ma, Guang Yang

**Affiliations:** ^1^ Shanghai Key Laboratory of Magnetic Resonance, East China Normal University, Shanghai, China; ^2^ Department of Radiology, Shengli Clinical Medical College of Fujian Medical University, Fujian Provincial Hospital, Fuzhou, China; ^3^ Magnetic Resonance (MR) Research Collaboration Team, Siemens Healthineers (China), Shanghai, China

**Keywords:** habitat, intravoxel incoherent motion (IVIM), breast cancer, immunohistochemistry, radiomics

## Abstract

**Background:**

To explore the value of intravoxel incoherent motion (IVIM)-based habitat imaging in predicting immunohistochemistry in patients with breast cancer.

**Methods:**

299 patients with suspected breast cancer were randomly assigned to a training set of 210 individuals and a test set of 89 individuals. A series of models was constructed for human epidermal growth factor receptor 2 (HER2)/Ki-67/hormone receptors (HR)/lymph node metastasis (LNM) prediction, including the whole-tumor model, habitat model, conventional MRI features (CF) model and hybrid model (incorporating habitats features and CF). The performance of various models was evaluated with the area under the receiver operating characteristic curve (AUC) and decision curve analysis (DCA). P (two-tailed) < 0.05 was considered statistically significant.

**Results:**

On the test cohort, for HER2/HR/LNM, the habitats model achieved the highest AUC values of 0.692/0.651/0.722, higher than those of the whole-tumor model (AUC = 0.591/0.599/0.609) and the CF model (AUC = 0.598/0.603/0.608). For Ki-67, the CF model achieved a highest AUC of 0.746. The hybrid model achieved AUC values of 0.706/0.762/0.668/0.728 for HER2/Ki67/HR/LNM. DeLong test showed a significant difference between habitats model and the whole-tumor model for LNM (*P* = 0.006).

**Conclusion:**

While habitat features can provide richer biological information, the models combining habitats and CF obtained more accurate results than other models, making them promising candidates for clinical application in breast cancer diagnosis.

## Introduction

Breast cancer ranks among the most common cancers impacting women globally (1). It is a diverse disease characterized by a range of histopathological traits, molecular classifications, and clinical patterns, necessitating ongoing research to enhance diagnostic accuracy, prognostic assessments, and therapeutic approaches (2, 3). Immunohistochemistry (IHC) plays a vital role in the characterization of breast cancer, utilizing biomarkers such as hormone receptors (HR), human epidermal growth factor receptor 2 (HER2), Ki-67, and lymph node metastasis (LNM) to identify subtypes, predict prognosis, and assess treatment responsiveness (4). HR-positive cancers respond well to hormonal therapies, while HER2-positive cancers are more aggressive but can be targeted by drugs like trastuzumab. Ki-67 levels indicate tumor proliferation and LNM is crucial for staging and prognosis, influencing treatment strategies (5–8).

Magnetic resonance imaging (MRI) significantly improves the diagnosis, staging, and management of breast cancer by providing high-quality soft tissue contrast along with comprehensive functional insights (9). Intravoxel incoherent motion (IVIM) can obtain multiple quantitative parameters, such as the apparent pure diffusion coefficient (D), pseudo-diffusion coefficient (D*), and perfusion fraction (*f*) from multiple diffusion weighted imaging (DWI) images with different b-values using a biexponential fitting algorithm (10–12). These parameters provide non-invasively information about diffusion and perfusion simutaneously, allowing for a better characterization of breast cancer.

Radiomics extracts quantitative features from medical images, offering insights beyond what is visible to the human eye (13). MRI-based radiomics aids in tumor characterization and predicting treatment response (14). However, it is often challenging to relate radiomics features to biological or physiological meanings. A rule of thumb for radiomics study is that radiomics features should be extracted from a well-mixed region, but lesions of tumor may comprise of mesoscopically inhomogeneous regions of different characteristics, limiting its potential for medical research and treatment decision-making (14, 15).

Habitat analysis in radiomics addresses tumor heterogeneity by analyzing different regions, or “habitats”, within the tumor (16). This method takes into account the spatial complexity of tumors and offers valuable insights into their biological characteristics (17). IVIM imaging provides valuable parameters that describe both perfusion and diffusion within each voxel, while diffusion indicates the integrity and density of the cellular structure (18). Consequently, subregions clustered by IVIM parameters correspond to regions with different physiological characteristics. For example, regions with high cell density and high perfusion may correspond to areas of active tumor growth, whereas regions with low cell density and low perfusion may correspond to less aggressive or necrotic tissue (19). At the same time, integrating habitat features with clinical factors may contribute to a more comprehensive understanding of the biology of diseases and promote the development of precision medicine (20, 21). This study aims to explore the value of IVIM-based habitat imaging in predicting IHC in patients with breast cancer.

## Materials and methods

### Study setting and timeframe

**Figure 1:**
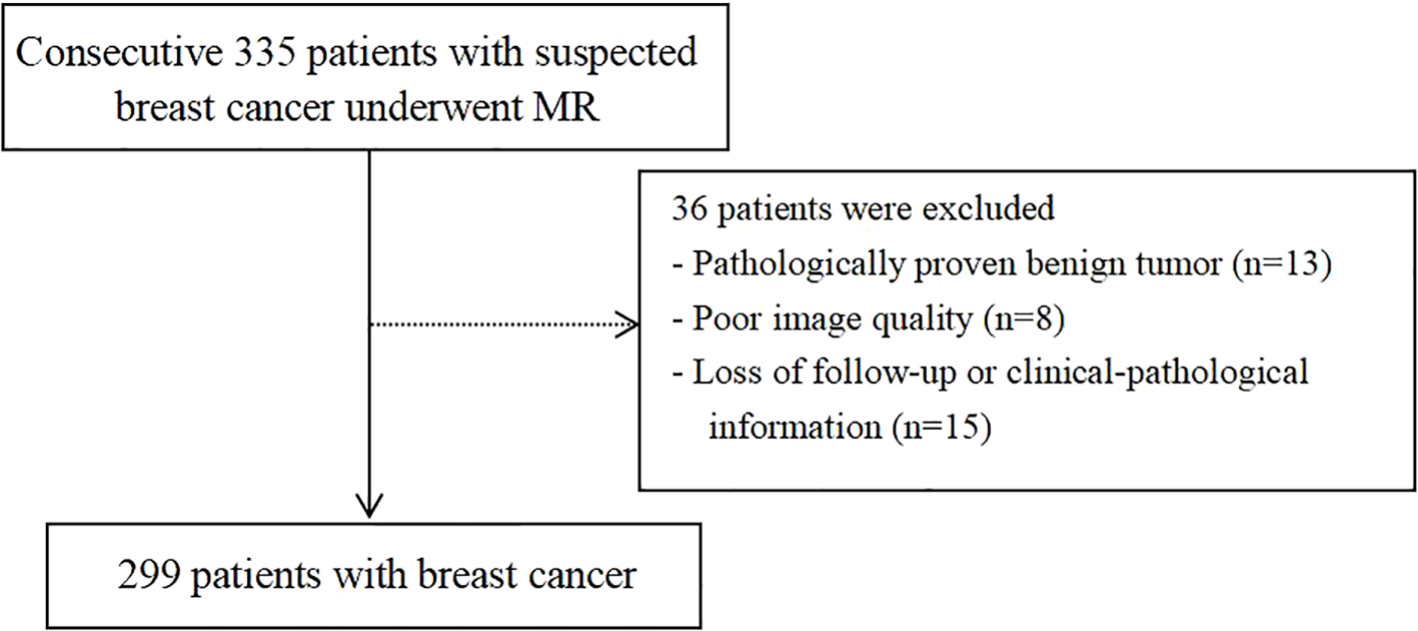
Flowchart depicting patient selection and grouping.

This retrospective study included patients with suspected breast cancer treated at Fujian Provincial Hospital from July 2019 to August 2023. The study protocol was approved by the hospital’s ethics committee (approval code: K2021-05-007, May 2019), and all methods adhered to relevant guidelines and regulations (22). Written informed consent to participate was obtained from all the patients. The inclusion criteria were: (I) no needle biopsy, radiotherapy, or chemotherapy before MRI examination; (II) availability of complete MRI review data with good image quality; (III) availability of complete pathological data; and (IV) without multicentric tumor. The process of patient selection and grouping has been illustrated in **Figure 1**.

**Figure 2:**
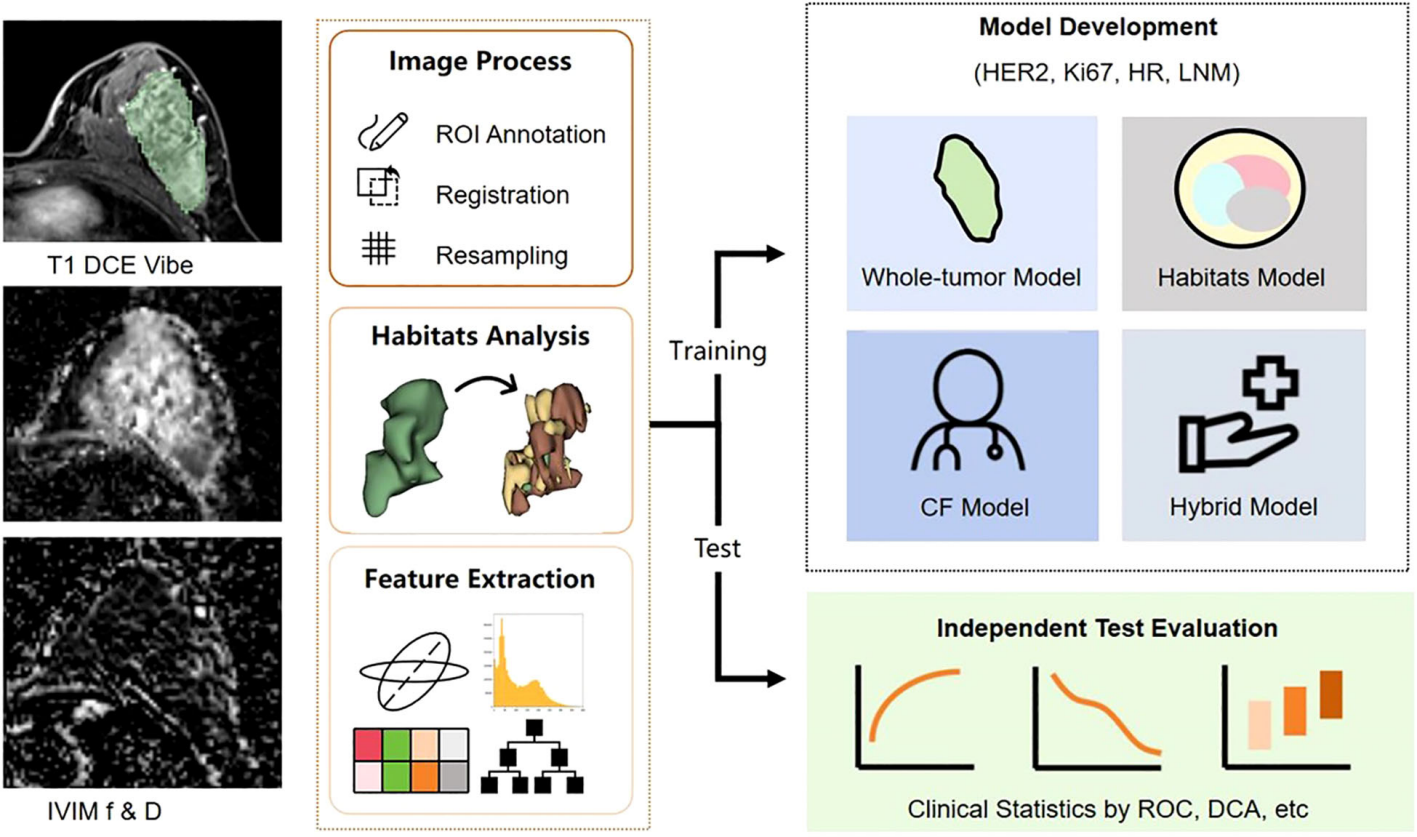
Workflow of the study.

The whole dataset was split into training and test cohorts at a 7:3 ratio. The training cohort was utilized for constructing the diagnostic model, while the test cohort was set aside for model evaluation. The study workflow was illustrated in **Figure 2**.

### Immunohistochemical analysis

HR-positive status was defined as ≥1% of tumor cell nuclei staining positively for either ER or PR, while HR-negative status was indicated by <1% positivity for both. Tumors with an HER2 membrane immunostaining score of 3+ were classified as HER2-positive, whereas a score of 2+ necessitated *in situ* hybridization to confirm HER2 amplification. Ki67 positivity required ≥30% of tumor cell nuclei to stain positively for Ki67. LNM positivity was defined as the presence of cancer cells in one or more lymph nodes, as determined through histopathological examination. Tissue examination was conducted by a pathologist (Y.H.) with two decades of expertise in breast tumor diagnosis.

### Imaging studies

MRI scans were conducted using a 3T scanner (MAGNETOM Prisma, Siemens Healthcare, Erlangen, Germany). To reduce noise and minimize anxiety-related motion, patients were provided earplugs prior to the examination. Scanning was conducted in a prone position, allowing the breasts to rest naturally within the coil. Detailed sequence parameters are provided in **Supplementary Table S1**. For the contrast-enhanced scans, patients were administered gadopentetate meglumine (Magnevist, 0.2 mmol/kg; GE Healthcare). A high-pressure syringe was used to inject the contrast agent into a dorsal hand vein at a flow rate of 1.5–2.0 mL/s, followed by a flush with 15–20 mL of normal saline to remove any remaining agent.

Voxel-wise fitting of diffusion-weighted imaging data was performed to produce IVIM maps, incorporating parameters D, f, and D*, using the Body Diffusion Toolbox software (Siemens Healthcare, Erlangen, Germany). The IVIM parameters D, f, and D* were estimated using a segmented fitting approach. Based on previous studies, a b-value threshold of 200 s/mm² was used to separate the perfusion and diffusion components (11). The D parameter was first obtained by linear fitting of the logarithmic signal at b-values above 200 s/mm². Subsequently, D and the full signal were used in a nonlinear biexponential fitting to estimate f and D*.

### Conventional MRI features

The tumor on the MR images was evaluated and annotated based on the 2013 Breast Imaging - Reporting and Data System (BI-RADS) guidelines for MRI. This was done by two radiologists: M.H., with 14 years of experience in breast imaging, and Y.Z., with 5 years of experience. They focused on conventional MRI features (CF), such as fibroglandular tissue (FGT), background parenchymal enhancement (BPE), high T2 signal, mass shape, mass margin, internal enhancement pattern, non-mass internal enhancement pattern, architectural distortion, and time-intensity curve.

### Tumor segmentation

The IVIM images were analyzed using 3D Slicer (v4.10.2, www.slicer.org) for segmentation. The first radiologist, M.H., with 14 years of experience in breast imaging, performed the segmentation by outlining a three-dimensional volume of interest (VOI) that covered the solid tumor component, based on dynamic contrast-enhanced MRI scans. A second radiologist, Y.Z., with 5 years of experience, independently segmented 30 randomly chosen tumors from the training set. The repeated VOIs were used to evaluate the feature robustness.

### Habitat analysis and feature extraction

For whole-tumor analysis, we used the open-source FAE (v0.5.7) based on PyRadiomics to extract features from the VOI in images of each sequence (23). These radiomic features include shape, first order, and texture features based on gray-level co-occurrence matrix (GLCM), gray-level size zone matrix (GLSZM), gray-level run length matrix (GLRLM), neighborhood gray tone difference matrix (NGTDM) and gray-level dependence matrix (GLDM). The specific image preprocessing procedures include Z-score normalization and equal frequency discretization. The complete feature extraction procedure is performed following the guidelines provided by the imaging biomarker standardization initiative (IBSI) to ensure that the extracted features are reproducible (**Supplementary Methods**) (24).

For habitat analysis, the intensity of D and *f* of each voxel in the VOI was combined into a two-dimensional vector, and K-means clustering, an unsupervised clustering method, was used to cluster all vectors in the VOI into clusters. By assigning all voxels in the same cluster to a same subregion, the whole VOI was divided into subregions. To determine the optimal K value for K-means clustering, we utilized the calinski-harabasz (CH) Score (25) (**Supplementary 1**). For each subregion within the VOI, we calculated its volume and volume fraction, and extracted first-order histogram features from D-map and f-map.

### Feature selection and model construction

Based on the original whole-tumor features, habitat features, and CF, we developed four types of IHC models. After conducting feature selection and model training, we constructed the whole-tumor model, habitat model, and CF model to predict each type of IHC. Further, we combined the habitats features and the CF features to construct the hybrid model.

To enhance model robustness, interobserver reproducibility was evaluated using the two-way random absolute agreement intraclass correlation coefficient (ICC). Features with an ICC below 0.75 were excluded, retaining only those deemed stable.

We normalized the features in the training set using Z-scores and eliminated redundant features based on the Pearson correlation coefficient (PCC). For pairs of features with a PCC greater than 0.99, one was randomly removed. For whole tumor radiomics models utilizing a large number of features, feature selection was conducted using least absolute shrinkage and selection operator (LASSO) regression, where the alpha parameter was set between 5–^3^ to 5–^2^ (26). For the final model construction, we combined four feature selection algorithms and two classifiers, selecting the optimal model based on the highest cross-validation area under the curve (AUC) during 5-fold cross-validation on the training cohort. The feature selection algorithms employed were recursive feature elimination (RFE), the Kruskal-Wallis (KW) test, analysis of variance (ANOVA) and Relief, while the classifiers used were support vector machine (SVM) and logistic regression (LR). Additional details are provided in **Supplementary 2** and **Supplementary 3**.

### Statistical analysis

Statistical analysis was conducted using SPSS v28.0.1.1. The normality of continuous variables was tested with the Shapiro-Wilk test, while Levene’s test was used to assess homogeneity of variance. Variables with a normal distribution were expressed as mean ± standard deviation and compared using the independent samples t-test. For non-normally distributed data, variables were reported as median (interquartile range) and analyzed using the Mann-Whitney U test. Categorical data were presented as frequency (percentage) and compared using the Pearson chi-square test or the continuity-corrected chi-square test. The predictive performance of the models was evaluated through receiver operating characteristic (ROC) curves and several classification metrics, including AUC, sensitivity (Sen), specificity (Spe), positive predictive value (PPV), negative predictive value (NPV), accuracy (Acc), and Matthews correlation coefficient (MCC). Decision curve analysis (DCA) was conducted to visualize the net benefit rate versus the threshold for IHC prediction. Feature importance was evaluated using the Shapley Additive Explanations (SHAP) method to assess the contribution of each feature to the model’s predictions. DeLong test was performed to statistically compare the models and a *post hoc* power analysis was conducted to assess whether the sample size was sufficient to detect a meaningful difference in model performance. A p-value of less than 0.05 (two-tailed) was considered statistically significant.

## Results

### Characteristics of the study sample

Based on the selection criteria, 299 women diagnosed with breast cancer were included in this study. The patients were randomly assigned to a training set of 210 individuals and a test set of 89 individuals. No significant differences (p > 0.05) in IHC results were found between the two sets (**Table 1**).

**Table 1:** Characteristics of patients in the training and test sets (n = 299).

CF/IHC	Training set (n = 210)	Test set (n = 89)	*P* value
Age	50.1 ± 10.2	50.7 ± 12.1	0.657
FGT			0.541
a/b	176 (84)	72 (81)	
c/d	34 (16)	17 (19)	
BPE			0.822
a/b	95 (45)	39 (44)	
c/d	115 (55)	50 (56)	
High T2 signal			0.867
Positive	57 (27)	25 (28)	
Negative	153 (73)	64 (72)	
Masses	139 (66)	56 (63)	0.587
Mass shape (%)			0.040
Round/Oval	33 (24)	6 (11)	
Irregular	106 (76)	50 (89)	
Mass margin (%)			0.240
Spiculated	69 (50)	33 (59)	
Non-spiculated	70 (50)	23 (41)	
Internal enhancement pattern (%)			0.379
Rim enhancement	84 (60)	30 (54)	
No rim enhancement	55 (40)	26 (46)	
Non-mass enhancement	71 (34)	33 (37)	
Non-mass internal enhancement pattern (%)			0.176
Clustered ring	60 (85)	31 (94)	
Homogeneous/Heterogeneous	11 (15)	2 (6)	
Architectural distortion			0.037
Positive	39 (19)	8 (9)	
Negative	171 (81)	81 (91)	
Time-intensity curve			0.867
I	57 (27)	25 (28)	
II/III	153 (73)	64 (72)	
HER2 status, n (%)			0.845
Positive	67 (32)	28 (31)	
Negative	143 (68)	61 (69)	
Ki67 index, n (%)			0.080
High	101 (48)	33 (37)	
Low	109 (52)	56 (63)	
HR status, n (%)			0.174
Positive	144 (69)	68 (76)	
Negative	66 (31)	21 (24)	
LNM status, n (%)			0.667
Positive	117 (56)	52 (58)	
Negative	93 (44)	37 (42)	

CF, conventional magnetic resonance imaging features; IHC, immunohistochemistry; FGT, fibroglandular tissue; BPE, background parenchymal enhancement; FGT: a. Almost entirely fat b. Scattered fibroglandular tissue c. Heterogeneous fibroglandular tissue d. Extreme fibroglandular tissue; BPE: a. Minimal b. Mild c. Moderate d. Marked; Time-intensity curve: I. Persistent II. Plateau III. Washout; HR, hormone receptor; HER2, human epidermal growth factor receptor 2; LNM, lymph node metastasis.

### Habitat clustering

**Figure 3:**
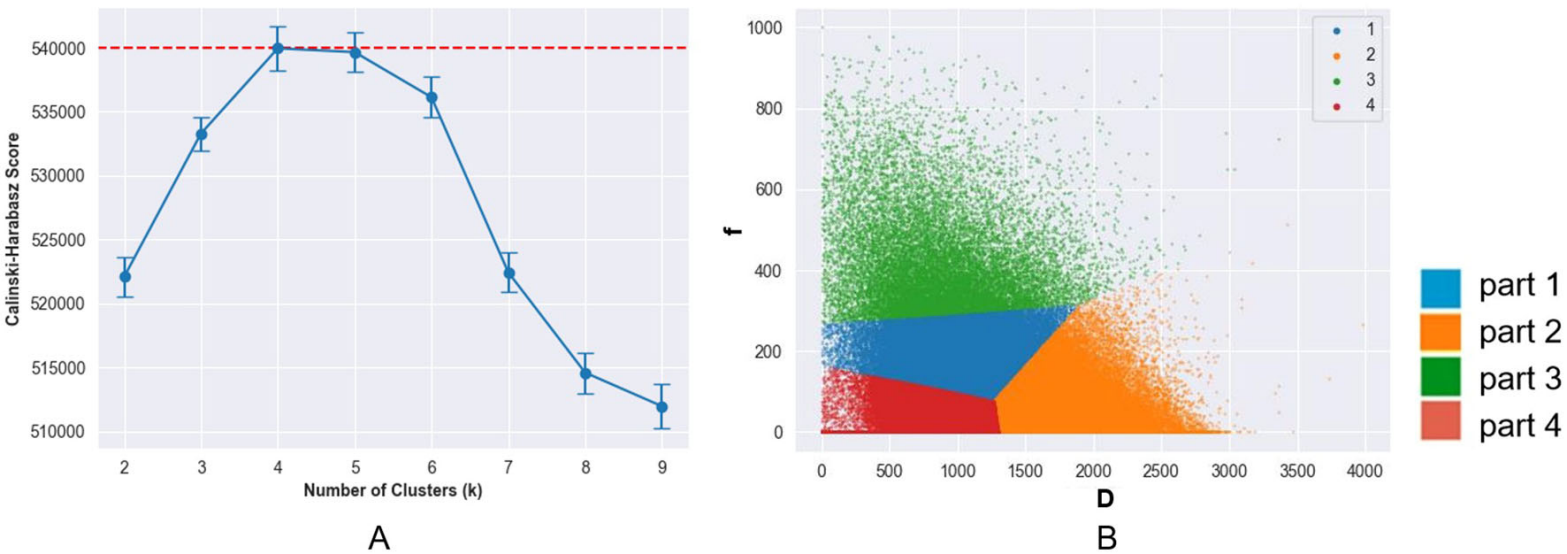
**(a)** Change of calinski-harabasz (CH) score with number of clusters (K) values. The CH score reached its peak at K = 4, indicating the optimal number of clusters. **(b)** Voxel distribution in volume of interest in the D and f maps (K = 4). The tumors were subdivided into four subregions: a low D-value region (part 1), a high D-value region (part 2), a high f-value region (part 3), and a region with both low D-value and low f-value (part 4).

According to the CH index, the best clustering result was achieved when K=4 (**Figure 3a**), which means that the tumor was split into four regions: Part-1 with low D and mediate *f*, Part-2 with high D, Part-3 with high *f*, and Part-4 with low D and low *f*. (**Figure 3b**)

### Performance of the models and model comparison

**Figure 4:**
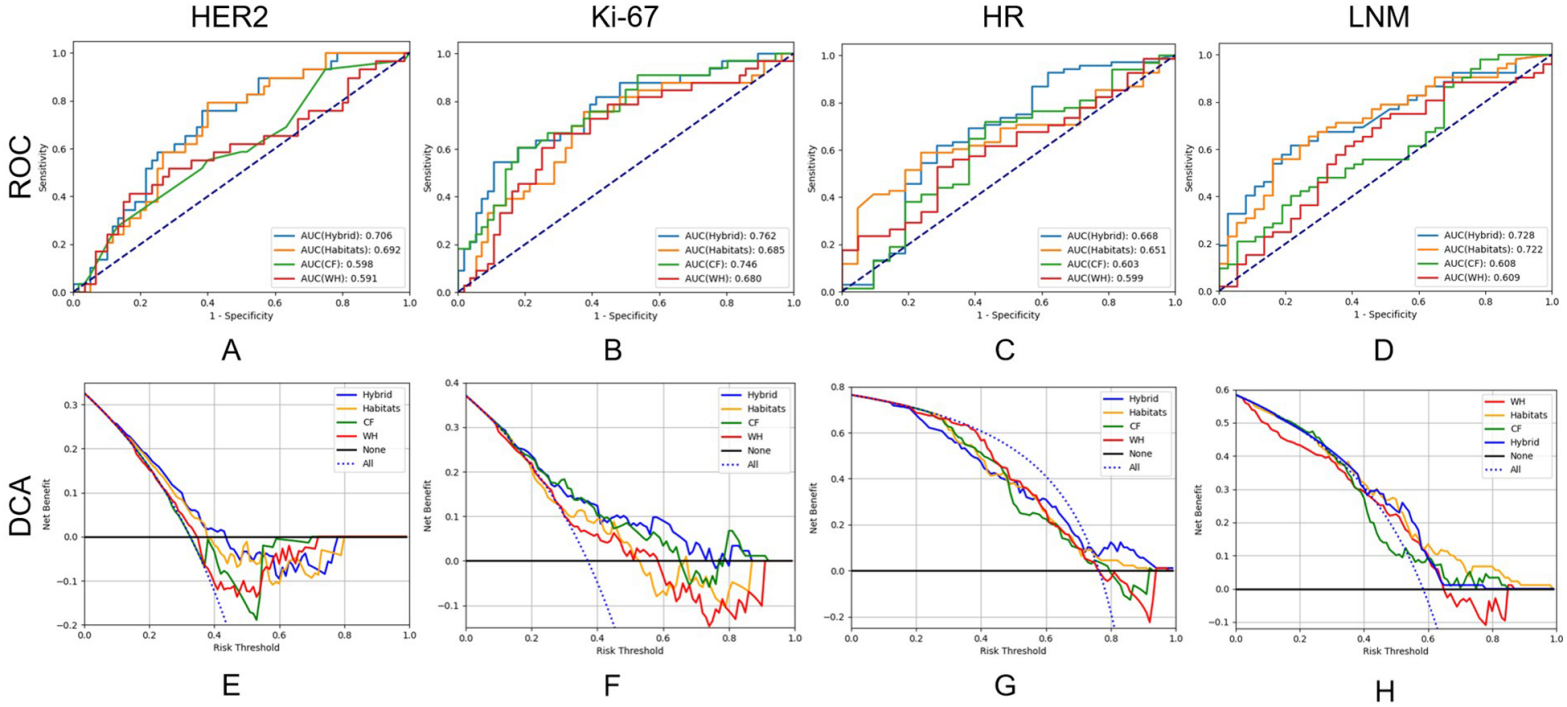
Receiver operating characteristic curves and decision curve analysis of the performance of all models on test cohort.

**Figure 5:**
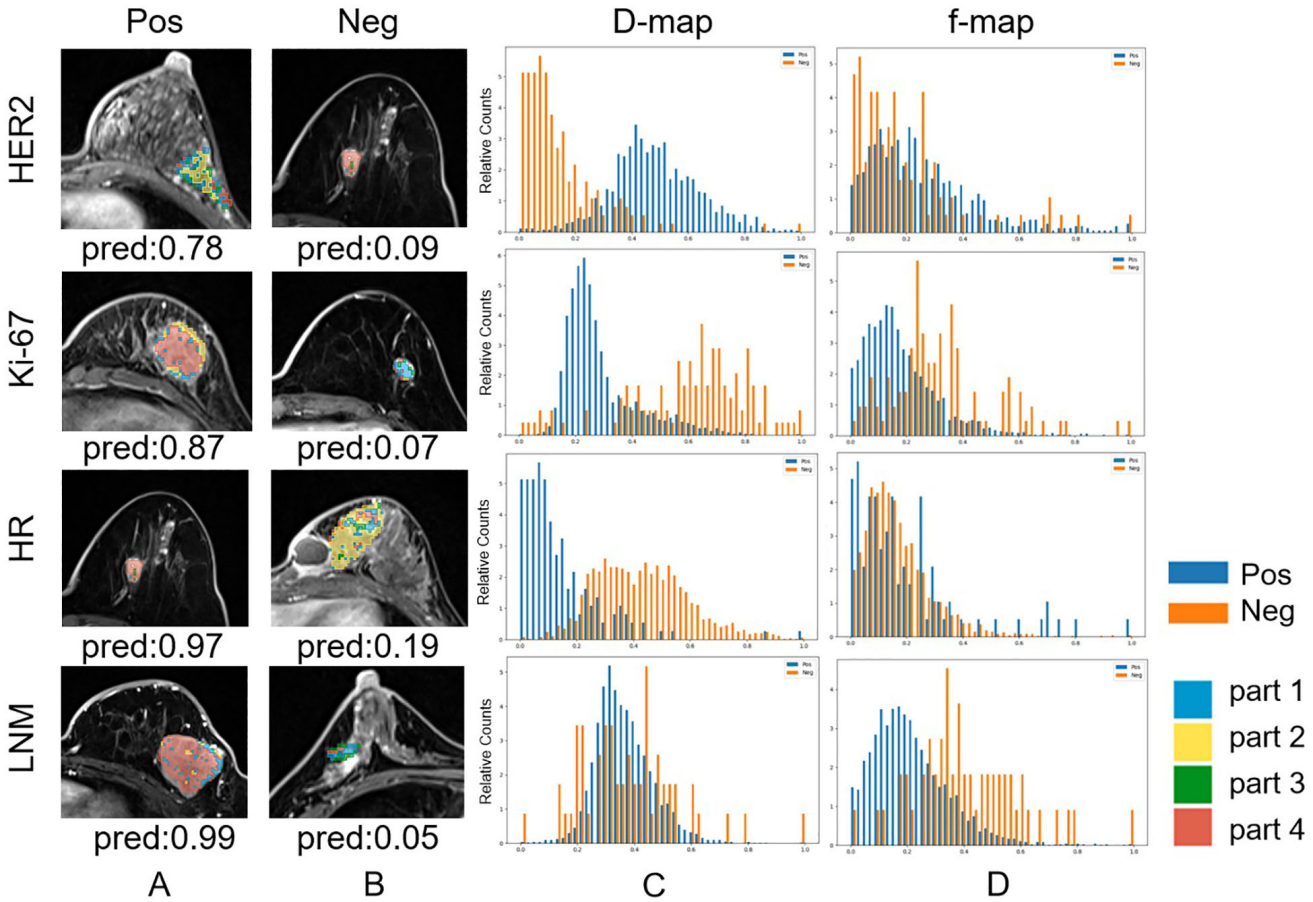
Habitat imaging and histogram of correctly predicted cases whose predicted probabilities are closest to their ground truth labels (Note that the best results for HER2-Neg and HR-Pos prediction occur in the same case). **(a)** Habitat imaging of HER2/Ki-67/HR/LNM positive cases. For HER2/Ki-67/HR/LNM, voxels in positive lesions are predominantly in part 2/4/4/4. **(b)** Habitat imaging of HER2/Ki-67/HR/LNM negative cases. For HER2/Ki-67/HR/lymph node metastasis, voxels in negative lesions are predominantly in part 4/1/2/2,3. **(c)** Histogram of D values for both HER2/Ki-67/HR/LNM positive and negative cases. **(d)** Histogram of F values for both HER2/Ki-67/HR/LNM positive and negative cases.

We constructed the whole-tumor model, habitat model, CF model and hybrid model for HER2/Ki67/HR/LNM. The performance of all models is outlined in **Table 2**. ROC curves and DCA curves of all models were shown in **Figure 4**. Histogram analysis of the predicted case of the habitat model is shown in **Figure 5**.

**Table 2:** Performance of models over the test cohort.

IHC	Model	AUC (95% CI)	Acc	Sen	Spe	NPV	PPV	MCC
HER2	Whole-tumor	0.591 (0.458, 0.725)	0.584	0.586	0.583	0.745	0.405	0.159
	Habitat	0.692 (0.581, 0.803)	0.584	0.793	0.483	0.829	0.426	0.265
	CF	0.598 (0.472, 0.724)	0.584	0.517	0.617	0.726	0.395	0.127
	**Hybrid**	**0.706 (0.596, 0.816)**	**0.584**	**0.828**	**0.467**	**0.849**	**0.429**	**0.286**
Ki67	Whole-tumor	0.680 (0.562, 0.798)	0.652	0.697	0.625	0.778	0.523	0.311
	Habitat	0.685 (0.567, 0.803)	0.640	0.451	0.750	0.700	0.517	0.211
	CF	0.746 (0.640, 0.853)	0.700	0.667	0.714	0.784	0.579	0.372
	**Hybrid**	**0.762 (0.658, 0.867)**	**0.697**	**0.636**	**0.732**	**0.774**	**0.583**	**0.363**
HR	Whole-tumor	0.599 (0.467, 0.732)	0.573	0.559	0.619	0.302	0.826	0.151
	Habitat	0.651 (0.531, 0.771)	0.551	0.471	0.810	0.321	0.889	0.242
	CF	0.603 (0.455, 0.751)	0.685	0.838	0.191	0.267	0.770	0.033
	**Hybrid**	**0.668 (0.518, 0.819)**	**0.629**	**0.618**	**0.667**	**0.350**	**0.857**	**0.243**
LNM	Whole-tumor	0.609 (0.487, 0.731)	0.629	0.692	0.541	0.556	0.679	0.234
	Habitat	0.722 (0.615, 0.829)	0.640	0.789	0.432	0.593	0.661	0.237
	CF	0.608 (0.488, 0.728)	0.629	0.692	0.541	0.556	0.679	0.234
	**Hybrid**	**0.728 (0.624, 0.832)**	**0.685**	**0.615**	**0.784**	**0.592**	**0.800**	**0.396**

AUC, area under the curve; CI, confidence interval; Acc, accuracy; Sen, sensitivity; Spe, specificity; NPV, negative predictive value; PPV, positive predictive value; MCC, matthews correlation coefficient.

*Bold face indicates the models with the highest AUC values for the specific tasks.

For HER2, the habitats model achieved an AUC of 0.692 (95% CI: 0.581-0.803), higher than those of the whole-tumor model (AUC = 0.591, 95% CI: 0.458-0.725) and the CF model (AUC = 0.598, 95% CI: 0.472-0.724). The hybrid model achieved an AUC of 0.706 (95% CI: 0.596-0.816), but not significantly higher than the habitats model. **Figure 4e** shows that the net benefits of hybrid model were higher than other models when the threshold was in the range of 0.2-0.6, but when the threshold value was above 0.6, CF models provides more benefits.

For Ki-67, the CF model achieved an AUC of 0.746 (95% CI: 0.640-0.853), higher than those of the whole-tumor model (AUC = 0.680, 95% CI: 0.562-0.798) and habitats model (AUC = 0.685, 95% CI: 0.567-0.803). The hybrid model achieved the highest AUC of 0.762 (95% CI: 0.658-0.867). **Figure 4f** shows that the net benefit of hybrid model was higher than other models when the threshold was in the range of 0.4-0.8.

For HR, the AUC of habitats model (0.651, 95% CI: 0.531-0.771) was higher than the whole-tumor model (AUC = 0.599, 95% CI: 0.467-0.732) and the CF model (AUC = 0.603, 95% CI: 0.455-0.751). The hybrid model achieved an AUC of 0.668 (95% CI: 0.518-0.819), but not significantly higher than the habitats model. **Figure 4g** shows that the net benefit of hybrid model was higher than other models when the threshold was in the range of 0.6-1.0.

For LNM, the habitats model achieved an AUC of 0.722 (95% CI: 0.615-0.829), significantly (*p* < 0.05) higher than the whole-tumor model (AUC = 0.609, 95% CI: 0.487-0.731) and CF model (AUC = 0.608, 95% CI: 0.488-0.728). The hybrid model achieved an AUC of 0.728 (95% CI: 0.624-0.832). **Figure 4h** shows that the net benefit of habitats model was higher than other models when the threshold was in the range of 0.6-1.0.

**Figure 6:**
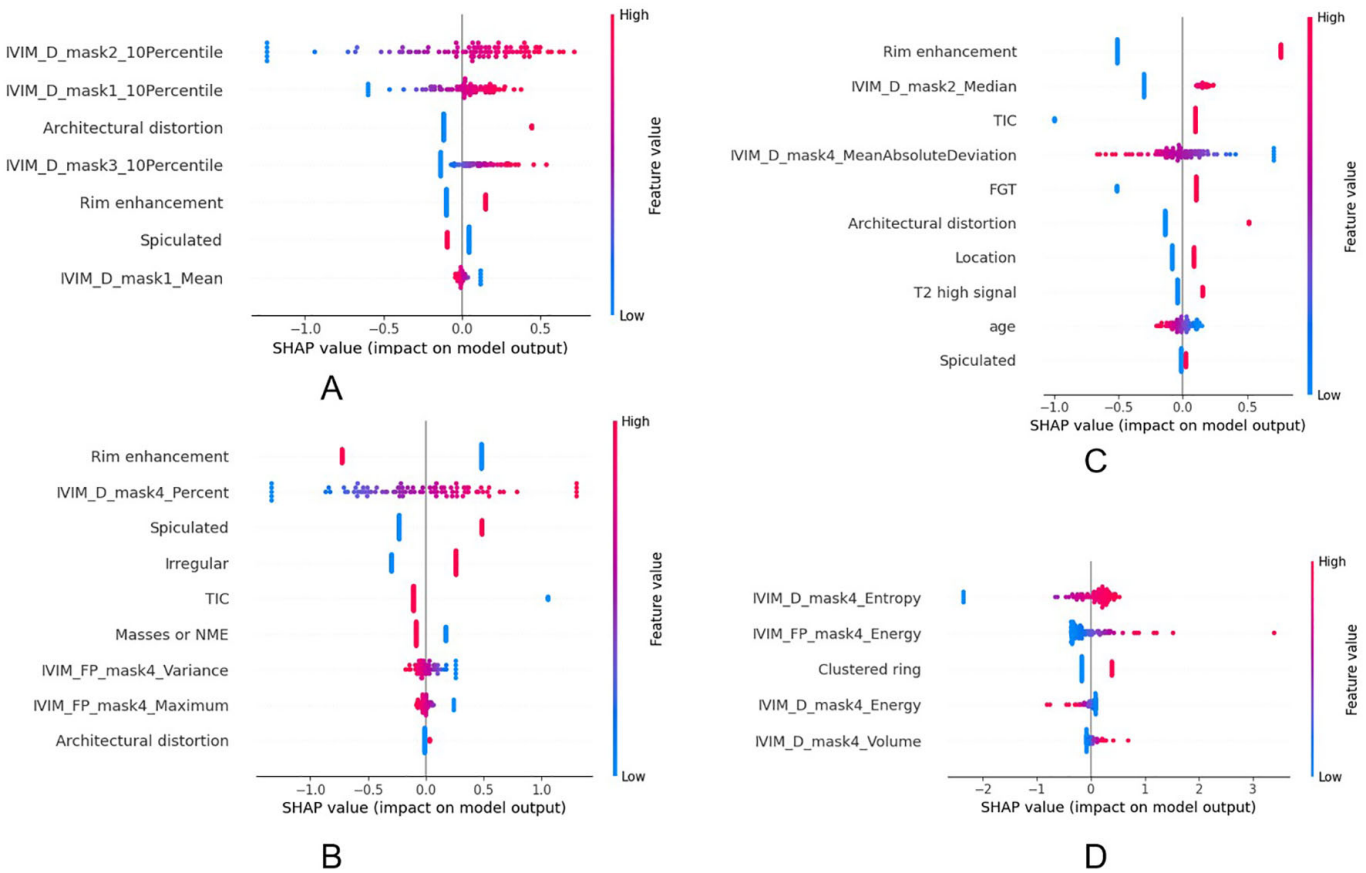
The impact of each feature on the hybrid model’s predictions in HER2 **(a)**, HR **(b)**, Ki67 **(c)** and LNM **(d)**.

The SHAP visualization is shown in **Figure 6**. The feature details for all models are provided in **Supplementary Tables S2**-**S5**, the model performance on the training set is presented in **Supplementary Table S6**, the DeLong test results for the different models are included in **Supplementary Table S7**, and the power analysis results are included in **Supplementary Table S8**.

## Discussion

In this study, we explored the features from the whole tumor, habitats region and clinical characters, and found that the hybrid model combining habitat features and CF performs best in the prediction of these four IHC.

Compared to whole-tumor analysis, more refined habitat VOIs can better reflect tumor heterogeneity. In our study, the tumor was subdivided into four subregions: Part-1 (low D, moderate *f*) likely represents regions of high cellular density with preserved perfusion. Part-2 (high D) suggests regions of reduced cellularity, where elevated water diffusion (high D) aligns with necrotic or edematous zones (27). Part-3 (high *f*) reflects hyperperfused subregions, indicative of active angiogenesis (28). These areas may correlate with highly vascularized tumor fronts or inflammatory microenvironments, often associated with rapid growth or immune infiltration. Part-4 (low D, low *f*) denotes densely packed, hypoxic niches with restricted diffusion and poor perfusion. Such habitats are histologically consistent with high-grade tumors, which drive metastasis and chemoresistance (29). By analyzing these subregions, our study provided a more in-depth understanding of tumor heterogeneity.

Our study revealed several distinct associations between IVIM-derived habitat features and breast cancer molecular subtypes. In the high-D and low-*f* subregion (Part-2), a higher 10th percentile value of D was associated with HER2-positive tumors. This suggests that in HER2-positive tumors, even the most diffusion-restricted voxels within this subregion exhibit relatively higher diffusivity. Such a pattern may reflect a structurally loose and infiltrative growth pattern characteristic of HER2-positive breast cancers, where even the densest parts of low-perfusion regions are less compact. This finding aligns with the known aggressive and spatially invasive behavior of HER2-positive tumors (30). Furthermore, in the same subregion (Part-2), a higher median value of D was associated with Ki-67 positivity. This may be related to the rapid proliferation of tumors with high Ki67 and the occurrence of necrosis. Such regions may correspond to loosely structured stromal areas or infiltrative tumor margins, where low cellular packing density allows for increased water mobility despite reduced vascularization. This finding may reflect a distinct tumor microenvironment in highly proliferative breast cancers, characterized by rapid cellular turnover in structurally less constrained regions (31, 32). We also found that the proportion of tumor volume characterized by both D and low f (Part-4) was significantly higher in HR-positive tumors. This may reflect the relatively slow-growing and fibrotic nature of HR-positive tumors, which tend to accumulate more structurally stable, hypoperfused, and diffusion-restricted regions (33). In contrast, HR-negative tumors, including triple-negative breast cancer (TNBC), are often more heterogeneous and rapidly proliferative, resulting in fragmented or necrotic cores with unstable D/*f* patterns (34, 35). In the low-D and low-f subregion (Part-4), both D entropy and *f* energy were found to be significantly associated with LNM status. A higher entropy of D reflects increased heterogeneity in water diffusion, potentially indicating a complex microenvironment comprising necrosis, inflammation, fibrosis, and heterogeneous cell density. Such microstructural complexity has been correlated with more aggressive tumor behavior and higher metastatic potential (36). Similarly, a higher energy of *f* in the same region suggests the presence of repetitive, spatially organized perfusion signals, possibly representing residual microcirculation or neovascular structures within necrotic or fibrotic areas (37, 38). The elevated energy of perfusion-related features in low-perfusion regions underscores the role of organized microvascular structures in supporting tumor progression and metastasis. In addition, among clinical features, rim enhancement showed a notable association with Ki-67 positivity and HR negativity. Rim enhancement is characterized by peripheral contrast uptake with central hypoenhancement on DCE-MRI, often reflecting a combination of central necrosis and peripheral angiogenesis (39). This enhancement pattern is more frequently observed in tumors with high Ki-67 expression, as rapid tumor proliferation tends to outpace central vascular supply, leading to necrotic cores surrounded by viable, actively growing tumor rims (40). Furthermore, rim enhancement is commonly associated with hormone receptor-negative breast cancers, especially TNBC (41).

It is worth noting that habitat models constructed in this study used only first-order histogram features, which greatly improved the interpretability of the model. Besides, first-order histogram features are influenced only by the intensity values of the voxels, leading to greater stability in the analysis (42). This inherent stability enhances the robustness and reproducibility of habitat models, making them less susceptible to noise and variations in image acquisition parameters (43). In contrast, traditional radiomics used a bunch of texture features, which are not only difficult to interpret, but also readily influenced by image preprocessing steps, including image discretization and re-segmentation. Furthermore, the hybrid model, constructed with clinical and habitat features, improved the performance without compromising the interpretability, thus could be more readily accepted by radiologists.

The clinical relevance of IVIM-based habitat imaging lies in its ability to non-invasively map tumor heterogeneity by identifying subregions with distinct biological characteristics. By clustering voxels based on D and *f* parameters, habitat imaging can differentiate areas of high cellularity, necrosis, or active angiogenesis within tumors. This spatial resolution offers a deeper understanding of tumor biology, which is critical for predicting immunohistochemical markers such as HER2, Ki-67, HR, and LNM. These insights could guide clinicians in tailoring treatment strategies, such as prioritizing HER2-targeted therapies for tumors with high-D habitats or intensifying surveillance for patients with low-D/low-*f* subregions suggestive of aggressive behavior. Integrating habitat imaging into clinical workflows could enhance diagnostic accuracy and therapeutic planning. For example, during preoperative MRI evaluations, habitat maps could help surgeons target biopsy sites to regions of high proliferative activity, improving diagnostic yield. In radiation oncology, identifying hypoxic or perfusion-deficient subregions might enable dose escalation to radioresistant zones. Additionally, habitat features could supplement existing BI-RADS criteria to refine risk stratification, potentially reducing unnecessary interventions. The hybrid model, which combines habitat features with conventional MRI characteristics, further improves predictive performance, offering radiologists a tool to automate IHC predictions during routine image interpretation. This could streamline decision-making in settings where rapid biomarker assessment is critical, such as neoadjuvant therapy monitoring. Despite these advantages, several barriers may hinder clinical adoption. Technical standardization remains a challenge, as variations in MRI protocols (e.g., b-value selection, IVIM fitting algorithms) across institutions could compromise reproducibility. The clinical integration of IVIM-based habitat imaging requires a structured, multi-phase approach to ensure feasibility and reliability. First, standardization of MRI acquisition protocols is critical. This involves consensus-driven guidelines for b-value selection, IVIM fitting algorithms, and segmentation methodologies. At the same time, it is important to develop user-friendly software that enables automated habitat clustering and feature extraction. Integrating such tools into existing PACS or AI platforms could help streamline clinical workflows. These tools should emphasize interpretability by offering radiologists intuitive visualizations, such as color-coded habitat maps overlaid on MRI images, along with quantitative summaries like subregion volumes or perfusion metrics to support clinical decision-making. As habitat imaging remains in a developmental phase, the external validity of IVIM-based habitat models continues to be uncertain, thereby impeding their integration into standard clinical workflows.

However, there are also several limitations in our study that need to be acknowledged. The relatively small sample size (n = 299), with only 210 cases used for training, raises concerns regarding potential overfitting. Although internal cross-validation was performed to mitigate this risk, such an approach may not fully reflect the model’s generalizability to unseen data. Future studies should incorporate larger, multicenter datasets and external validation cohorts to better evaluate the robustness and clinical applicability of the model across different populations and imaging conditions. Additionally, we did not explore the specifics of IVIM reconstruction parameters. For example, the selection of b-values and the approach used to fit diffusion and perfusion components. While intraclass correlation coefficients (ICCs >0.75) were used to ensure feature robustness, segmentation discrepancies between radiologists, particularly in heterogeneous tumors with ill-defined margins, could affect habitat clustering and feature extraction. Furthermore, large-scale pathological studies, including animal experiments, may be needed to reflect the global spatial consistency of tumors.

In conclusion, compared with traditional radiomics models, the model combining habitats and CF features can achieve better performance and better interpretability. It can potentially help to improve the image-based diagnosis, and help doctors formulate personalized treatment plans for breast cancer patients. Habitat radiomics based on IVIM can help to better understand the biology of tumors, providing more information and support for clinical decision-making. Future research will further improve and validate the models to further improve their applicability and effectiveness in clinical settings.

## Data Availability

The original contributions presented in the study are included in the article/**Supplementary Material**. Further inquiries can be directed to the corresponding author.
