# Supplementary material for: Intravoxel incoherent motion-based habitat imaging for the prediction of immunohistochemistry in patients with breast cancer
Source: Front Oncol. 2025 Jun 27;15:1595157. doi: 10.3389/fonc.2025.1595157 (PMC12245771; doi:10.3389/fonc.2025.1595157)
Supplement: Supplementary file 1 [file Table1.docx]

**Table S1.** Imaging protocol of MR sequences

|  | TR | TE | Thickness | Slices | Bandwidth | Matrix | Averages | Concatenations | FOV | Others |
| --- | --- | --- | --- | --- | --- | --- | --- | --- | --- | --- |
| Fat saturation T2WI | 3739 ms | 69 ms | 4 mm | 35 | 246 Hz/Px | 384 × 384 | 2 | 2 | 340 mm × 340 mm | / |
| T1WI | 6.03 ms | 2.82 ms | 0.9 mm | 160 | 300 Hz/Px | 403 × 448 | 1 | 1 | 340 mm × 340 mm | / |
| DCE-MRI | 4.03 ms | 1.33 ms | 1.5 mm | 112 | 1120 Hz/Px | 259 × 320 | 1 | 1 | 350 mm × 350 mm | Measurements 36 |
| DWI | 5700 ms | 62 ms | 4 mm | 35 | 2024 Hz/Px | 114 × 190 | 1, 2, 2, 2, 2, 2, 2, 2, and 3 | 1 | 340 mm × 340 mm | B-values 0, 30, 50, 80, 120, 160, 200, 500, and 1000 s/mm^2^ |
|  |  |  |  |  |  |  |  |  |  |  |
|  | Turbo  /EPI  Fctor | TI | Flip  angle | Dimension | Trajectory | PAT mode | Phase oversampling | Correction | Scan time |  |
| Fat saturation T2WI | 16 | 230ms | 80 deg | 2D | Cartesian | GRAPPA | 10% | Prescan Normalize | 157s |  |
| T1WI | / | / | 12 deg | 3D | Cartesian | GRAPPA | 29% | Prescan Normalize | 117s |  |
| DCE-MRI | / | / | 10 deg | 2D | Cartesian | CAIPIRINHA | 28.6% | Prescan Normalize | 343 s |  |
| DWI | 116 | / | / | 2D | Cartesian | GRAPPA | 30% | Prescan Normalize | 218 s |  |

**Table S2.** Feature details for the HER2 model

| Starting feature set | Final feature set (weight) |
| --- | --- |
| **Whole-tumor (number = 3358)** | **(number = 2)** |
| Shape features, first-order features, second-order features, and texture features, including GrayLevelCooccurence Matrix (GLCM), Gray LevelRun Length Matrix (GLRLM), Gray LevelSize Zone Matrix (GLSZM), Neighbouring Gray Tone Difference Matrix (NGTDM), Gray Level Dependence Matrix (GLDM) from D and F maps | IVIM-D_exponential_gldm_DependenceEntropy (0.428)  IVIM-D_gradient_glcm_Imc1 (0.298) |
| **Habitat (number = 156)** | **(number = 4)** |
| D_mask and f_mask  (10Percentile, 90Percentile, Energy, Entropy, InterquartileRange, Kurtosis, Maximum, MeanAbsoluteDeviation, Mean, Median, Minimum, Range, RobustMeanAbsoluteDeviation, RootMeanSquared, Skewness, TotalEnergy, Uniformity, Variance, Volume)  IVIM-D (IVIM-f)_mask_Percent | IVIM-D_mask1_10Percentile (0.235)  IVIM-D_mask1_Mean (-0.179)  IVIM-D_mask3_10Percentile (0.147)  IVIM-D_mask2_10Percentile (0.560) |
| **Conventional MRI features (number = 12)** | **(number = 5)** |
| Age  FGT  BPE level  Location  Masses or NME  Irregular  Spiculated  Rim enhancement  Clustered ring  Architectural distortion  T2 high signal  TIC | Spiculated (-0.048)  Rim enhancement (0.326)  Clustered ring (0.265)  Architectural distortion (0.227)  T2 high signal (-0.224) |
| **Hybrid (number = 9)** | **(number = 7)** |
| IVIM-D_mask1_10Percentile  IVIM-D_mask1_Mean  IVIM-D_mask3_10Percentile  IVIM-D_mask2_10Percentile  Spiculated  Rim enhancement  Clustered ring  Architectural distortion  T2 high signal | IVIM-D_mask1_10Percentile (0.204)  IVIM-D_mask1_Mean (-0.034)  IVIM-D_mask3_10Percentile (0.147)  IVIM-D_mask2_10Percentile (0.459)  Spiculated (-0.066)  Rim enhancement (0.123)  Architectural distortion (0.219) |

**Table S3.** Feature details for the Ki67 model

| Starting feature set | Final feature set (weight) |
| --- | --- |
| **Whole-tumor (number = 3358)** | **(number = 2)** |
| Shape features, first-order features, second-order features, and texture features, including GrayLevelCooccurence Matrix (GLCM), Gray LevelRun Length Matrix (GLRLM), Gray LevelSize Zone Matrix (GLSZM), Neighbouring Gray Tone Difference Matrix (NGTDM), Gray Level Dependence Matrix (GLDM) from D and F maps | IVIM-D_original_firstorder_Skewness (0.550)  IVIM-D_squareroot_firstorder_Skewness (0.445) |
| **Habitat (number = 156)** | **(number = 7)** |
| D_mask and f_mask  (10Percentile, 90Percentile, Energy, Entropy, InterquartileRange, Kurtosis, Maximum, MeanAbsoluteDeviation, Mean, Median, Minimum, Range, RobustMeanAbsoluteDeviation, RootMeanSquared, Skewness, TotalEnergy, Uniformity, Variance, Volume)  IVIM-D (IVIM-f)_mask_Percent | IVIM-D_mask1_Skewness (0.249)  IVIM-D_mask2_Median (0.455)  IVIM-D_mask4_MeanAbsoluteDeviation (0.174)  IVIM-D_mask4_Minimum (0.139)  IVIM-D_mask4_Skewness (0.619)  IVIM-D_mask4_Variance (-0.201)  IVIM-FP_mask1_Kurtosis (0.156) |
| **Conventional MRI features (number = 12)** | **(number = 12)** |
| Age  FGT  BPE level  Location  Masses or NME  Irregular  Spiculated  Rim enhancement  Clustered ring  Architectural distortion  T2 high signal  TIC | Age (-0.129)  FGT (0.216)  BPE level (0.036)  Location (0.074)  Masses or NME (0.167)  Irregular (0.314)  Spiculated (-0.266)  Rim enhancement (0.454)  Clustered ring (-0.048)  Architectural distortion (0.328)  T2 high signal (0.080)  TIC (0.291) |
| **Hybrid (number = 19)** | **(number = 10)** |
| IVIM-D_mask1_Skewness  IVIM-D_mask2_Median  IVIM-D_mask4_MeanAbsoluteDeviation  IVIM-D_mask4_Minimum  IVIM-D_mask4_Skewness  IVIM-D_mask4_Variance  IVIM-FP_mask1_Kurtosis  Age  FGT  BPE level  Location  Masses or NME  Irregular  Spiculated  Rim enhancement  Clustered ring  Architectural distortion  T2 high signal  TIC | IVIM-D_mask2_Median (0.220)  IVIM-D_mask4_MeanAbsoluteDeviation (-0.263)  Age (-0.069)  FGT (0.227)  Location (0.085)  Spiculated (0.018)  Rim enhancement (0.629)  Architectural distortion (0.254)  T2 high signal (0.087)  TIC (0.290) |

**Table S4.** Feature details for the HR model

| Starting feature set | Final feature set (weight) |
| --- | --- |
| **Whole-tumor (number = 3358)** | **(number = 13)** |
| Shape features, first-order features, second-order features, and texture features, including GrayLevelCooccurence Matrix (GLCM), Gray LevelRun Length Matrix (GLRLM), Gray LevelSize Zone Matrix (GLSZM), Neighbouring Gray Tone Difference Matrix (NGTDM), Gray Level Dependence Matrix (GLDM) from D and F maps | IVIM-D_original_firstorder_10Percentile (-0.521)  IVIM-D_original_firstorder_90Percentile (-0.353)  IVIM-D_original_firstorder_Mean (0.005)  IVIM-D_square_firstorder_10Percentile (-0.293)  IVIM-D_square_firstorder_Mean (0.248)  IVIM-D_square_firstorder_RootMeanSquared (0.206)  IVIM-D_squareroot_firstorder_10Percentile (-0.479)  IVIM-D_squareroot_firstorder_90Percentile (-0.321)  IVIM-D_squareroot_firstorder_Mean (-0.055)  IVIM-D_squareroot_firstorder_Median (0.518)  IVIM-D_wavelet-LLL_firstorder_90Percentile (-0.957)  IVIM-D_wavelet-LLL_firstorder_Median (0.558)  IVIM-D_wavelet-LLL_firstorder_RootMeanSquared (0.082) |
| **Habitat (number = 156)** | **(number = 4)** |
| D_mask and f_mask  (10Percentile, 90Percentile, Energy, Entropy, InterquartileRange, Kurtosis, Maximum, MeanAbsoluteDeviation, Mean, Median, Minimum, Range, RobustMeanAbsoluteDeviation, RootMeanSquared, Skewness, TotalEnergy, Uniformity, Variance, Volume)  IVIM-D (IVIM-f)_mask_Percent | IVIM-D_mask4_Percent (0.572)  IVIM-FP_mask4_Maximum (-0.099)  IVIM-FP_mask4_Variance (1.185)  IVIM-FP_mask4_MeanAbsoluteDeviation (-1.328) |
| **Conventional MRI features (number = 12)** | **(number = 12)** |
| Age  FGT  BPE level  Location  Masses or NME  Irregular  Spiculated  Rim enhancement  Clustered ring  Architectural distortion  T2 high signal  TIC | Age (-0.016)  FGT (0.016)  BPE level (0.063)  Location (0.028)  Masses or NME (-0.552)  Irregular (0.343)  Spiculated (0.455)  Rim enhancement (-0.450)  Clustered ring (-0.519)  Architectural distortion (-0.007)  T2 high signal (-0.260)  TIC (-0.258) |
| **Hybrid (number = 16)** | **(number = 9)** |
| IVIM_D_mask4_Percent  IVIM_FP_mask4_Maximum  IVIM_FP_mask4_Variance  IVIM_FP_mask4_MeanAbsoluteDeviation  Age  FGT  BPE level  Location  Masses or NME  Irregular  Spiculated  Rim enhancement  Clustered ring  Architectural distortion  T2 high signal  TIC | IVIM-D_mask4_Percent (0.539)  IVIM-FP_mask4_Maximum (-0.057)  IVIM-FP_mask4_Variance (-0.075)  Masses or NME (-0.122)  Irregular (0.279)  Spiculated (0.341)  Rim enhancement (-0.595)  Architectural distortion (0.017)  TIC (-0.309) |

**Table S5.** Feature details for the LNM model

| Starting feature set | Final feature set (weight) |
| --- | --- |
| **Whole-tumor (number = 3358)** | **(number = 9)** |
| Shape features, first-order features, second-order features, and texture features, including GrayLevelCooccurence Matrix (GLCM), Gray LevelRun Length Matrix (GLRLM), Gray LevelSize Zone Matrix (GLSZM), Neighbouring Gray Tone Difference Matrix (NGTDM), Gray Level Dependence Matrix (GLDM) from D and F maps | IVIM-D_square_glcm_Idn (-0.333)  IVIM-D_square_gldm_HighGrayLevelEmphasis (-0.813)  IVIM-D_wavelet-LHL_glcm_ClusterTendency (0.117)  IVIM-D_wavelet-LHL_glcm_Idm (0.030)  IVIM-D_wavelet-LHL_glcm_Idmn (-0.127)  IVIM-D_wavelet-LHL_glcm_Idn (-0.017)  IVIM-D_wavelet-LHL_glcm_InverseVariance (1.089)  IVIM-D_wavelet-LHL_glcm_SumSquares (0.015)  IVIM-D_wavelet-LHL_glrlm_GrayLevelVariance (0.240) |
| **Habitat (number = 156)** | **(number = 4)** |
| D_mask and f_mask  (10Percentile, 90Percentile, Energy, Entropy, InterquartileRange, Kurtosis, Maximum, MeanAbsoluteDeviation, Mean, Median, Minimum, Range, RobustMeanAbsoluteDeviation, RootMeanSquared, Skewness, TotalEnergy, Uniformity, Variance, Volume)  IVIM-D (IVIM-f)_mask_Percent | IVIM-D_mask4_Energy (-0.037)  IVIM-D_mask4_Entropy (0.486)  IVIM-D_mask4_Volume (0.184)  IVIM-FP_mask4_Energy (0.798) |
| **Conventional MRI features (number = 12)** | **(number = 12)** |
| Age  FGT  BPE level  Location  Masses or NME  Irregular  Spiculated  Rim enhancement  Clustered ring  Architectural distortion  T2 high signal  TIC | Age (-0.360)  FGT (0.022)  BPE level (-0.475)  Location (0.027)  Masses or NME (-0.264)  Irregular (0.187)  Spiculated (0.159)  Rim enhancement (0.137)  Clustered ring (0.438)  Architectural distortion (0.424)  T2 high signal (-0.181)  TIC (-0.268) |
| **Hybrid (number = 16)** | **(number = 5)** |
| IVIM_D_mask4_Percent  IVIM_FP_mask4_Maximum  IVIM_FP_mask4_Variance  IVIM_FP_mask4_MeanAbsoluteDeviation  Age  FGT  BPE level  Location  Masses or NME  Irregular  Spiculated  Rim enhancement  Clustered ring  Architectural distortion  T2 high signal  TIC | IVIM-D_mask4_Energy (-0.199)  IVIM-D_mask4_Entropy (0.516)  IVIM-D_mask4_Volume (0.162)  IVIM-FP_mask4_Energy (0.700)  Clustered ring (0.247) |

**Table S6.** Performance of models over the train cohort.

| **IHC** | **Model** | **AUC** (95% CI) | **Acc** | **Sen** | **Spe** | **NPV** | **PPV** | **MCC** |
| --- | --- | --- | --- | --- | --- | --- | --- | --- |
| HER2 | Whole-tumor | 0.613 (0.534, 0.692) | 0.586 | 0.652 | 0.556 | 0.777 | 0.402 | 0.192 |
|  | Habitat | 0.629 (0.550, 0.707) | 0.586 | 0.742 | 0.514 | 0.813 | 0.412 | 0.240 |
|  | CF | 0.646 (0.569, 0.723) | 0.624 | 0.621 | 0.625 | 0.783 | 0.432 | 0.230 |
|  | Hybrid | 0.667 (0.591, 0.744) | 0.576 | 0.803 | 0.472 | 0.840 | 0.411 | 0.263 |
|  |  |  |  |  |  |  |  |  |
| Ki67 | Whole-tumor | 0.718 (0.648, 0.787) | 0.700 | 0.703 | 0.697 | 0.717 | 0.683 | 0.400 |
|  | Habitat | 0.725 (0.655, 0.792) | 0.705 | 0.564 | 0.835 | 0.674 | 0.760 | 0.416 |
|  | CF | 0.748 (0.681, 0.814) | 0.710 | 0.703 | 0.716 | 0.722 | 0.696 | 0.418 |
|  | Hybrid | 0.739 (0.669, 0.805) | 0.714 | 0.723 | 0.706 | 0.733 | 0.695 | 0.429 |
|  |  |  |  |  |  |  |  |  |
| HR | Whole-tumor | 0.737 (0.667, 0.808) | 0.657 | 0.583 | 0.818 | 0.474 | 0.875 | 0.374 |
|  | Habitat | 0.694 (0.617, 0.771) | 0.624 | 0.528 | 0.833 | 0.447 | 0.874 | 0.340 |
|  | CF | 0.684 (0.603, 0.765) | 0.752 | 0.903 | 0.424 | 0.667 | 0.774 | 0.380 |
|  | Hybrid | 0.701 (0.622, 0.780) | 0.695 | 0.688 | 0.712 | 0.511 | 0.839 | 0.374 |
|  |  |  |  |  |  |  |  |  |
| LNM | Whole-tumor | 0.776 (0.713, 0.839) | 0.729 | 0.735 | 0.720 | 0.684 | 0.768 | 0.454 |
|  | Habitat | 0.724 (0.655, 0.793) | 0.705 | 0.846 | 0.527 | 0.731 | 0.692 | 0.398 |
|  | CF | 0.694 (0.623, 0.765) | 0.643 | 0.479 | 0.850 | 0.564 | 0.800 | 0.346 |
|  | Hybrid | 0.717 (0.648, 0.786) | 0.695 | 0.778 | 0.591 | 0.679 | 0.705 | 0.377 |

AUC: area under the curve; CI: confidence interval; Acc, accuracy; Sen, sensitivity; Spe, specificity; NPV, negative predictive value; PPV, positive predictive value; MCC, matthews correlation coefficient.

**Table S7.** Comparison of models using DeLong test.

|  | **Whole-tumor** | **Habitat** | **CF** | **Hybrid** |
| --- | --- | --- | --- | --- |
| **HER2** |  |  |  |  |
| Whole-tumor | 1.0 | 0.212 | 0.943 | 0.184 |
| Habitat | 0.212 | 1.0 | 0.209 | 0.587 |
| CF | 0.943 | 0.209 | 1.0 | 0.103 |
| Hybrid | 0.184 | 0.587 | 0.103 | 1.0 |
|  |  |  |  |  |
| **Ki67** |  |  |  |  |
| Whole-tumor | 1.0 | 0.898 | 0.355 | 0.194 |
| Habitat | 0.898 | 1.0 | 0.386 | 0.214 |
| CF | 0.355 | 0.386 | 1.0 | 0.571 |
| Hybrid | 0.194 | 0.214 | 0.571 | 1.0 |
|  |  |  |  |  |
| **HR** |  |  |  |  |
| Whole-tumor | 1.0 | 0.416 | 0.968 | 0.354 |
| Habitat | 0.416 | 1.0 | 0.625 | 0.841 |
| CF | 0.968 | 0.625 | 1.0 | 0.196 |
| Hybrid | 0.354 | 0.841 | 0.196 | 1.0 |
|  |  |  |  |  |
| **LNM** |  |  |  |  |
| Whole-tumor | 1.0 | **0.006** | 0.995 | **0.017** |
| Habitat | **0.006** | 1.0 | 0.114 | 0.743 |
| CF | 0.995 | 0.114 | 1.0 | 0.081 |
| Hybrid | **0.017** | 0.743 | 0.081 | 1.0 |

* Bold face indicates a statistically significant difference between the two models (*p* < 0.05).

**Table S8.** Results of statistical power analysis.

|  | **Whole-tumor** | **Habitat** | **CF** | **Hybrid** |
| --- | --- | --- | --- | --- |
| **HER2** |  |  |  |  |
| Whole-tumor |  | 29.3% | 5.1% | 36.7% |
| Habitat | 29.3% |  | 33.1% | 5.5% |
| CF | 5.1% | 33.1% |  | 26.1% |
| Hybrid | 36.7% | 5.5% | 26.1% |  |
|  |  |  |  |  |
| **Ki67** |  |  |  |  |
| Whole-tumor |  | 5.1% | 16.4% | 23.2% |
| Habitat | 5.1% |  | 14.8% | 21.1% |
| CF | 16.4% | 14.8% |  | 5.7% |
| Hybrid | 23.2% | 21.1% | 5.7% |  |
|  |  |  |  |  |
| **HR** |  |  |  |  |
| Whole-tumor |  | 11.1% | 5.0% | 16.0% |
| Habitat | 11.1% |  | 10.2% | 5.7% |
| CF | 5.0% | 10.2% |  | 14.7% |
| Hybrid | 16.0% | 5.7% | 14.7% |  |
|  |  |  |  |  |
| **LNM** |  |  |  |  |
| Whole-tumor |  | 36.3% | 5.0% | 39.8% |
| Habitat | 36.3% |  | 36.8% | 5.1% |
| CF | 5.0% | 36.8% |  | 40.3% |
| Hybrid | 39.8% | 5.1% | 40.3% |  |

**Supplement 1. The details of cluster number selection**

To assess the stability of the Calinski-Harabasz (CH) scores across different cluster numbers (K), we performed a bootstrap-based resampling strategy to evaluate the stability of CH scores across different values of K. Specifically, we randomly resampled the dataset 50 times (with replacement), and computed the CH score for each bootstrap sample. The standard deviation of these scores was used as an estimate of the standard error (SE) for each K.

We then applied the One Standard Error rule, where we selected the smallest K whose CH score was greater than or equal to (CH_max − SE_max). This approach favors simpler and more stable models. As a result, the optimal number of clusters was identified as K=4, providing a balance between clustering performance and robustness.

**Supplement 2. The details of the criteria for feature selection**

For least absolute shrinkage and selection operator (LASSO), we defined the alpha range for LASSO as 5⁻³ ~ 5⁻² (i.e., 0.005 ~ 0.05). Smaller alpha (e.g., 0.005) implies weaker regularization (retaining more features), while larger alpha (e.g., 0.05) forces more coefficients to zero. This range suits medium-scale datasets to balance model complexity and overfitting. Using exponential spacing (e.g., 5**n) efficiently explores regularization strengths across orders of magnitude. We employed 5-fold CV via LassoCV (cv=5). Max_iter=1000 ensures numerical stability under relaxed convergence tolerance (tol), avoiding biased parameter selection due to non-convergence.

For recursive feature elimination (RFE), the parameter n_features_to_select=20 defines the desired final number range of features (1~20) to retain. RFE eliminates features iteratively until this target is reached. If the total number of input features is fewer than the target (e.g., only 15 input features), the algorithm automatically adjusts the target to the available maximum. The step=0.05 setting indicates that 5% of the remaining features are removed in each iteration. The elimination process terminates when the number of remaining features is less than or equal to n_features_to_select.

For the kruskal–wallis (KW) test, features are ranked by their F-values (Kruskal-Wallis statistic), which measure distribution differences across label groups. The top 1~20 features with the highest F-values are retained.

For relief, features are ranked by the absolute values of Relief weights (calculated via L2-norm distance and neighbor comparisons), and the top 1~20 features are retained.

For analysis of variance (ANOVA), features are ranked by ANOVA F-values (measuring mean differences across label groups), and the top 1~20 features are selected.
 These criteria ensured that selected features contributed meaningfully to model performance while reducing the risk of overfitting.

**Supplement 3. Detailed specific hyperparameter values and tuning strategy**

1. Hyperparameter Configuration and Tuning for LR
2. Key Parameters and Defaults

Regularization (C): Default 1.0

Solver (solver): Default saga (supports l1, l2, and elasticnet penalties)

Penalty Type (penalty): Default l2; options include l1, elasticnet

Convergence Tolerance (tol): Default 0.01

Max Iterations (max_iter): Default 100

1. Tuning Strategy

Cross-Validation: Use grid search to optimize:

-C: (0.01, 0.1, 1.0, 5)

-tol: (0.00003, 0.0003, 0.003)

-max_iter: (100, 200)

Evaluation Metric: Best validation set performance based on AUC.

1. Hyperparameter Configuration and Tuning for SVM
2. Key Parameters and Defaults

Kernel (kernel): Default linear; alternatives include rbf, poly, sigmoid.

Regularization (C): Default 1.0

Kernel-Specific Parameters:

-gamma (for rbf/poly/sigmoid): Default scale (i.e., 1 / (n_features * X.var()))

-degree (for poly): Default 3

Convergence Tolerance (tol): Default 1e-3

1. Tuning Strategy

Cross-Validation: Use grid search to optimize:

-Linear Kernel (kernel=’linear’): Optimize C as [0.1, 0.3, 1.0, 3.0].

-RBF Kernel (kernel=’rbf’): Jointly optimize gamma as [0.1, 0.3, 1.0, 3.0].

-Polynomial Kernel (kernel=’poly’): Test degree as [2, 3, 4].

Evaluation Metric: Best validation set performance based on AUC.

**Supplemental Methods.**

During the preprocessing phase, we initially employed a re-segmentation strategy, applying a restriction of 3 sigma for the variables D, f, and D*. Subsequently, we discretized the images into 16 bins, utilizing the entire training dataset. To enhance the extraction of texture features, we implemented a 2.5 D merge strategy. The feature extraction process encompassed a comprehensive range of metrics, including first-order statistics, shape features, gray-level co-occurrence matrix (GLCM), gray-level run length matrix (GLRM), gray-level size zone matrix (GLSZM), gray-level dependence matrix (GLDM), and neighboring gray tone difference matrix (NGTDM). Additionally, we employed a wavelet transform utilizing a Coiflet 1 filter to capture further high-dimensional features. The entire feature-extraction process was conducted according to the Imaging Biomarker Standardization Initiative. We self-checked the IBSI guidelines for reporting on radiomics studies, Checklist for Artificial Intelligence in Medical Imaging (CLAIM) and radiomics quality score as follows:

***IBSI guidelines for reporting on radiomics studies***

| **Topic** |  | **Item** | **Description** | **Y/N** |
| --- | --- | --- | --- | --- |
| **Patient** |  |  |  |  |
| Region of interest^^[[1]](#footnote-0)^^ |  | 1 | Describe the region of interest that is being imaged. | √ |
| Patient preparation |  | 2a | Describe specific instructions given to patients prior to image acquisition, e.g. fasting prior to imaging. | √ |
|  |  | 2b | Describe administration of drugs to the patient prior to image acquisition, e.g. muscle relaxants. | √ |
|  |  | 2c | Describe the use of specific equipment for patient comfort during scanning, e.g. ear plugs. |  |
| Radioactive tracer | PET, SPECT | 3a | Describe which radioactive tracer was administered to the patient, e.g. 18F-FDG. |  |
|  | PET, SPECT | 3b | Describe the administration method. |  |
|  | PET, SPECT | 3c | Describe the injected activity of the radioactive tracer at administration. |  |
|  | PET, SPECT | 3d | Describe the uptake time prior to image acquisition. |  |
|  | PET, SPECT | 3e | Describe how competing substance levels were controlled.^^[[2]](#footnote-1)^^ |  |
| Contrast agent |  | 4a | Describe which contrast agent was administered to the patient. | √ |
|  |  | 4b | Describe the administration method. | √ |
|  |  | 4c | Describe the injected quantity of contrast agent. | √ |
|  |  | 4d | Describe the uptake time prior to image acquisition. | √ |
|  |  | 4e | Describe how competing substance levels were controlled. |  |
| Comorbidities |  | 5 | Describe if the patients have comorbidities that affect imaging.^^[[3]](#footnote-2)^^ |  |
| **Acquisition^^[[4]](#footnote-3)^^** |  |  |  |  |
| Acquisition protocol |  | 6 | Describe whether a standard imaging protocol was used, and where its description may be found. | √ |
| Scanner type |  | 7 | Describe the scanner type(s) and vendor(s) used in the study. | √ |
| Imaging modality |  | 8 | Clearly state the imaging modality that was used in the study, e.g. CT, MRI. | √ |
| Static/dynamic scans |  | 9a | State if the scans were static or dynamic. | √ |
|  | Dynamic scans | 9b | Describe the acquisition time per time frame. | √ |
|  | Dynamic scans | 9c | Describe any temporal modelling technique that was used. | √ |
| Scanner calibration |  | 10 | Describe how and when the scanner was calibrated. |  |
| Patient instructions |  | 11 | Describe specific instructions given to the patient during acquisition, e.g. breath holding. | √ |
| Anatomical motion correction |  | 12 | Describe the method used to minimise the effect of anatomical motion. |  |
| Scan duration |  | 13 | Describe the duration of the complete scan or the time per bed position. | √ |
| Tube voltage | CT | 14 | Describe the peak kilo voltage output of the X-ray source. |  |
| Tube current | CT | 15 | Describe the tube current in mA. |  |
| Time-of-flight | PET | 16 | State if scanner time-of-flight capabilities are used during acquisition. |  |
| RF coil | MRI | 17 | Describe what kind RF coil used for acquisition, incl. vendor. | √ |
| Scanning sequence | MRI | 18a | Describe which scanning sequence was acquired. | √ |
|  | MRI | 18b | Describe which sequence variant was acquired. | √ |
|  | MRI | 18c | Describe which scan options apply to the current sequence, e.g. flow compensation, cardiac gating. | √ |
| Repetition time | MRI | 19 | Describe the time in ms between subsequent pulse sequences. | √ |
| Echo time | MRI | 20 | Describe the echo time in ms. | √ |
| Echo train length | MRI | 21 | Describe the number of lines in k-space that are acquired per excitation pulse. | √ |
| Inversion time | MRI | 22 | Describe the time in ms between the middle of the inverting RF pulse to the middle of the excitation pulse. | √ |
| Flip angle | MRI | 23 | Describe the flip angle produced by the RF pulses. | √ |
| Acquisition type | MRI | 24 | Describe the acquisition type of the MRI scan, e.g. 3D. | √ |
| k-space traversal | MRI | 25 | Describe the acquisition trajectory of the k-space. | √ |
| Number of averages/ excitations | MRI | 26 | Describe the number of times each point in k-space is sampled. | √ |
| Magnetic field strength | MRI | 27 | Describe the nominal strength of the MR magnetic field. | √ |
| **Reconstruction^^[[5]](#footnote-4)^^** |  |  |  |  |
| In-plane resolution |  | 28 | Describe the distance between pixels, or alternatively the field of view and matrix size. | √ |
| Image slice thickness |  | 29 | Describe the slice thickness. | √ |
| Image slice spacing |  | 30 | Describe the distance between image slices.^^[[6]](#footnote-5)^^ |  |
| Convolution kernel | CT | 31a | Describe the convolution kernel used to reconstruct the image. |  |
|  | CT | 31b | Describe settings pertaining to iterative reconstruction algorithms. |  |
| Exposure | CT | 31c | Describe the exposure (in mAs) in slices containing the region of interest. |  |
| Reconstruction method | PET | 32a | Describe which reconstruction method was used, e.g. 3D OSEM. |  |
|  | PET | 32b | Describe the number of iterations for iterative reconstruction. |  |
|  | PET | 32c | Describe the number of subsets for iterative reconstruction. |  |
| Point spread function modelling | PET | 33 | Describe if and how point-spread function modelling was performed. |  |
| Image corrections | PET | 34a | Describe if and how attenuation correction was performed. |  |
|  | PET | 34b | Describe if and how other forms of correction were performed, e.g. scatter correction, randoms correction, dead time correction etc. |  |
| Reconstruction method | MRI | 35a | Describe the reconstruction method used to reconstruct the image from the k-space information. | √ |
|  | MRI | 35b | Describe any artifact suppression methods used during reconstruction to suppress artifacts due to undersampling of k-space. | √ |
| Diffusion-weighted imaging | DWI-MRI | 36 | Describe the b-values used for diffusion-weighting. | √ |
| **Image registration** |  |  |  |  |
| Registration method |  | 37 | Describe the method used to register multi-modality imaging. | √ |
| **Image processing - data conversion** | | | |  |
| SUV normalisation | PET | 38 | Describe which standardised uptake value (SUV) normalisation method is used. |  |
| ADC computation | DWI-MRI | 39 | Describe how apparent diffusion coefficient (ADC) values were calculated. | √ |
| Other data conversions |  | 40 | Describe any other conversions that are performed to generate e.g. perfusion maps. | √ |
| **Image processing - post-acquisition processing** | | | |  |
| Anti-aliasing |  | 41 | Describe the method used to deal with anti-aliasing when down-sampling during interpolation. |  |
| Noise suppression |  | 42 | Describe methods used to suppress image noise. |  |
| Post-reconstruction smoothing filter | PET | 43 | Describe the width of the Gaussian filter (FWHM) to spatially smooth intensities. |  |
| Skull stripping | MRI (brain) | 44 | Describe method used to perform skull stripping. |  |
| Non-uniformity correction^^[[7]](#footnote-6)^^ | MRI | 45 | Describe the method and settings used to perform non-uniformity correction. | √ |
| Intensity normalisation |  | 46 | Describe the method and settings used to normalise intensity distributions within a patient or patient cohort. | √ |
| Other post-acquisition processing methods |  | 47 | Describe any other methods that were used to process the image and are not mentioned separately in this list. | √ |
| **Segmentation** |  |  |  |  |
| Segmentation method |  | 48a | Describe how regions of interest were segmented, e.g. manually. | √ |
|  |  | 48b | Describe the number of experts, their expertise and consensus strategies for manual delineation. | √ |
|  |  | 48c | Describe methods and settings used for semi-automatic and fully automatic segmentation. |  |
|  |  | 48d | Describe which image was used to define segmentation in case of multi-modality imaging. | √ |
| Conversion to mask |  | 49 | Describe the method used to convert polygonal or mesh-based segmentations to a voxel-based mask. |  |
| **Image processing - image interpolation** | | | |  |
| Interpolation method |  | 50a | Describe which interpolation algorithm was used to interpolate the image. |  |
|  |  | 50b | Describe how the position of the interpolation grid was defined, e.g. align by center. |  |
|  |  | 50c | Describe how the dimensions of the interpolation grid were defined, e.g. rounded to nearest integer. |  |
|  |  | 50d | Describe how extrapolation beyond the original image was handled. |  |
| Voxel dimensions |  | 51 | Describe the size of the interpolated voxels. |  |
| Intensity rounding | CT | 52 | Describe how fractional Hounsfield Units are rounded to integer values after interpolation. |  |
| **Image processing - ROI interpolation** | | | |  |
| Interpolation method |  | 53 | Describe which interpolation algorithm was used to interpolate the region of interest mask. |  |
| Partially masked voxels |  | 54 | Describe how partially masked voxels after interpolation are handled. |  |
| **Image processing - re-segmentation** | | | |  |
| Re-segmentation methods |  | 55 | Describe which methods and settings are used to re-segment the ROI intensity mask. |  |
| **Image processing - discretisation** | | | |  |
| Discretisation method^^[[8]](#footnote-7)^^ |  | 56a | Describe the method used to discretise image intensities. | √ |
|  |  | 56b | Describe the number of bins (FBN) or the bin size (FBS) used for discretisation. |  |
|  |  | 56c | Describe the lowest intensity in the first bin for FBS discretisation.^^[[9]](#footnote-8)^^ |  |
| **Image processing - image transformation** | | | |  |
| Image filter^^[[10]](#footnote-9)^^ |  | 57 | Describe the methods and settings used to filter images, e.g. Laplacian-of-Gaussian. |  |
| **Radiomics feature computation** | | | |  |
| Feature set |  | 58 | Describe which set of radiomics features is computed and refer to their definitions or provide these. | √ |
| IBSI compliance |  | 59 | State if the software used to extract the set of features is able to reproduce the IBSI feature reference values.^^[[11]](#footnote-10)^^ | √ |
| Robustness |  | 60 | Describe how robustness of the features was assessed, e.g. test-retest analysis. | √ |
| Software availability |  | 61 | Describe which software and version was used to compute features. | √ |
| **Radiomics feature computation - texture parameters** | | | |  |
| Texture matrix aggregation |  | 62 | Define how texture-matrix based features were computed from underlying texture matrices. |  |
| Distance weighting |  | 63 | Define how CM, RLM, NGTDM and NGLDM weight distances, e.g. no weighting. |  |
| CM symmetry |  | 64 | Define whether symmetric or asymmetric co-occurrence matrices were computed. |  |
| CM distance |  | 65 | Define the (Chebyshev) distance at which co-occurrence of intensities is determined, e.g. 1. |  |
| SZM linkage distance |  | 66 | Define the distance and distance norm for which voxels with the same intensity are considered to belong to the same zone for the purpose of constructing an SZM, e.g. Chebyshev distance of 1. |  |
| DZM linkage distance |  | 67 | Define the distance and distance norm for which voxels with the same intensity are considered to belong to the same zone for the purpose of constructing a DZM, e.g. Chebyshev distance of 1. |  |
| DZM zone distance norm |  | 68 | Define the distance norm for determining the distance of zones to the border of the ROI, e.g. Manhattan distance. |  |
| NGTDM distance |  | 69 | Define the neighbourhood distance and distance norm for the NGTDM, e.g. Chebyshev distance of 1. |  |
| NGLDM distance |  | 70 | Define the neighbourhood distance and distance norm for the NGLDM, e.g. Chebyshev distance of 1. |  |
| NGLDM coarseness |  | 71 | Define the coarseness parameter for the NGLDM, e.g. 0. |  |
| **Machine learning and radiomics analysis** | | | |  |
| Diagnostic and prognostic modelling |  | 72 | See the TRIPOD guidelines for reporting on diagnostic and prognostic modelling. | √ |
| Comparison with known factors |  | 73 | Describe where performance of radiomics models is compared with known (clinical) factors. | √ |
| Multicollinearity |  | 74 | Describe where the multicollinearity between radiomics features in the signature is assessed. |  |
| Model availability |  | 75 | Describe where radiomics models with the necessary pre-processing information may be found. | √ |
| Data availability |  | 76 | Describe where imaging data and relevant meta-data used in the study may be found. | √ |

***Checklist for Artificial Intelligence in Medical Imaging (CLAIM)***

| **Section / Topic** | **No.** | **Item** | **Page / Line** | **No** | **NA** |
| --- | --- | --- | --- | --- | --- |
| **TITLE** / **ABSTRACT** |  |  |  |  |  |
|  | **1** | Identification as a study of AI methodology, specifying the category of technology used (e.g., deep learning) | **√** |  |  |
| **ABSTRACT** |  |  |  |  |  |
|  | **2** | Summary of study design, methods, results, and conclusions | **√** |  |  |
| **INTRODUCTION** |  |  |  |  |  |
|  | **3** | Scientific and/or clinical background, including the intended use and role of the AI approach | **√** |  |  |
|  | **4** | Study aims, objectives, and hypotheses | **√** |  |  |
| **METHODS** |  |  |  |  |  |
| ***Study Design*** | **5** | Prospective or retrospective study | **√** |  |  |
|  | **6** | Study goal | **√** |  |  |
| ***Data*** | **7** | Data sources | **√** |  |  |
|  | **8** | Inclusion and exclusion criteria | **√** |  |  |
|  | **9** | Data pre-processing | **√** |  |  |
|  | **10** | Selection of data subsets | **√** |  |  |
|  | **11** | De-identification methods | **√** |  |  |
|  | **12** | How missing data were handled | **√** |  |  |
|  | **13** | Image acquisition protocol | **√** |  |  |
| ***Reference Standard*** | **14** | Definition of method(s) used to obtain reference standard | **√** |  |  |
|  | **15** | Rationale for choosing the reference standard | **√** |  |  |
|  | **16** | Source of reference standard annotations | **√** |  |  |
|  | **17** | Annotation of test set | **√** |  |  |
|  | **18** | Measures of inter- and intra-rater variability of features described by the annotators | **√** |  |  |
| ***Data Partitions*** | **19** | How data were assigned to partitions | **√** |  |  |
|  | **20** | Level at which partitions are disjoint | **√** |  |  |
| ***Testing Data*** | **21** | Intended sample size | **√** |  |  |

| **Section / Topic** | **No.** | **Item** | **Page / Line** | **No** | **NA** |
| --- | --- | --- | --- | --- | --- |
| ***Model*** | **22** | Detailed description of model | **√** |  |  |
|  | **23** | Software libraries, frameworks, and packages | **√** |  |  |
|  | **24** | Initialization of model parameters | **√** |  |  |
| ***Training*** | **25** | Details of training approach | **√** |  |  |
|  | **26** | Method of selecting the final model | **√** |  |  |
|  | **27** | Ensembling techniques | **√** |  |  |
| ***Evaluation*** | **28** | Metrics of model performance | **√** |  |  |
|  | **29** | Statistical measures of significance and uncertainty | **√** |  |  |
|  | **30** | Robustness or sensitivity analysis | **√** |  |  |
|  | **31** | Methods for explainability or interpretability | **√** |  |  |
|  | **32** | Evaluation on internal data | **√** |  |  |
|  | **33** | Testing on external data |  | **√** |  |
|  | **34** | Clinical trial registration | **√** |  |  |
| **RESULTS** |  |  |  |  |  |
| ***Data*** | **35** | Numbers of patients or examinations included and excluded | **√** |  |  |
|  | **36** | Demographic and clinical characteristics of cases in each partition | **√** |  |  |
| ***Model performance*** | **37** | Performance metrics and measures of statistical uncertainty | **√** |  |  |
|  | **38** | Estimates of diagnostic performance and their precision | **√** |  |  |
|  | **39** | Failure analysis of incorrect results |  |  | **√** |
| **DISCUSSION** |  |  |  |  |  |
|  | **40** | Study limitations | **√** |  |  |
|  | **41** | Implications for practice, including intended use and/or clinical role | **√** |  |  |
| **OTHER INFORMATION** |  |  |  |  |  |
|  | **42** | Provide a reference to the full study protocol or to additional technical details | **√** |  |  |
|  | **43** | Statement about the availability of software, trained model, and/or data | **√** |  |  |
|  | **44** | Sources of funding and other support; role of funders | **√** |  |  |

* Indicate page and/or line number for each checklist item that is present. NA = not applicable.

***Radiomics quality score***

Image protocol quality-well-documented image protocols (for example, contrast, slice thickness, energy, etc.) and/ or usage of public image protocols allow reproducibility/ replicability

☑ protocols well documented

□ public protocol used

□ none

Multiple segmentations-possible actions are: segmentation by different physicians/ algorithms/ software, perturbing segmentations by (random) noise, segmentation at different breathing cycles. Analyse feature robustness to segmentation variabilities

☑ yes

□ no

Phantom study on all scanners-detect inter-scanner differences and vendor-dependent features. Analyse feature robustness to these sources of variability

☑ yes

□ no

Imaging at multiple time points-collect images of individuals at additional time points. Analyse feature robustness to temporal variabilities (for example, organ movement, organ expansion/ shrinkage)

☑ yes

□ no

Feature reduction or adjustment for multiple testing-decreases the risk of overfitting. Overfitting is inevitable if the number of features exceeds the number of samples. Consider feature robustness when selecting features

☑ Either measure is implemented

□ Neither measure is implemented

Multivariable analysis with non radiomics features (for example, EGFR mutation) - is expected to provide a more holistic model. Permits correlating/ inferencing between radiomics and non radiomics features

☑ yes

□ no

Detect and discuss biological correlates-demonstration of phenotypic differences (possibly associated with underlying gene-protein expression patterns) deepens understanding of radiomics and biology

☑ yes

□ no

Cut-off analyse-determine risk groups by either the median, a previously published cut-off or report a continuous risk variable. Reduces the risk of reporting overly optimistic results

☑ yes

□ no

Discrimination statistics-report discrimination statistics (for example, C-statistic, ROC curve, AUC) and their statistical significance (for example, p-values, confidence intervals). One can also apply resampling method (for example, bootstrapping, cross-validation)

☑ a discrimination statistic and its statistical significance are reported

□ a resampling method technique is also applied

□ none

Calibration statistics-report calibration statistics (for example, Calibration-in-the-large/slope, calibration plots) and their statistical significance (for example, P-values, confidence intervals). One can also apply resampling method (for example, bootstrapping, cross-validation)

☑ a calibration statistic and its statistical significance are reported

□ a resampling method technique is applied

□ none

Prospective study registered in a trial database -provides the highest level of evidence supporting the clinical validity and usefulness of the radiomics biomarker

☑ yes

□ no

Validation-the validation is performed without retraining and without adaptation of the cut-off value, provides crucial information with regard to credible clinical performance

□ No validation

☑ validation is based on a dataset from the same institute

□ validation is based on a dataset from another institute

□ validation is based on two datasets from two distinct institutes

□ the study validates a previously published signature

□ validation is based on three or more datasets from distinct institutes

Comparison to 'gold standard'-assess the extent to which the model agrees with/is superior to the current 'gold standard' method (for example, TNM-staging for survival prediction). This comparison shows the added value of radiomics

☑ yes

□ no

Potential clinical utility-report on the current and potential application of the model in a clinical setting (for example, decision curve analysis).

☑ yes

□ no

Cost-effectiveness analysis-report on the cost-effectiveness of the clinical application (for example, QALYs generated)

☑ yes

□ no

Open science and data-make code and data publicly available. Open science facilitates knowledge transfer and reproducibility of the study

□ scans are open source

□ region of interest segmentations are open source

□ the code is open sourced

☑ radiomics features are calculated on a set of representative ROIs and the calculated features and representative ROIs are open source

Total score

27 (75.00%)

1. Also referred to as volume of interest. [↑](#footnote-ref-0)
2. An example is glucose present in the blood which competes with the uptake of 18F-FDG tracer in tumour tissue. To reduce competition with the tracer, patients are usually asked to fast for several hours and a blood glucose measurement may be conducted prior to tracer administration. [↑](#footnote-ref-1)
3. An example of a comorbidity that may affect image quality in 18F-FDG PET scans are type I and type II diabetes melitus, as well as kidney failure. [↑](#footnote-ref-2)
4. Many acquisition parameters may be extracted from DICOM header meta-data, or calculated from them. [↑](#footnote-ref-3)
5. Many reconstruction parameters may be extracted from DICOM header meta-data. [↑](#footnote-ref-4)
6. Spacing between image slicing is commonly, but not necessarily, the same as the slice thickness,. [↑](#footnote-ref-5)
7. Also known as bias-field correction. [↑](#footnote-ref-6)
8. Discretisation may be performed separately to create intensity-volume histograms. If this is indeed the case, this should be described as well. [↑](#footnote-ref-7)
9. This is typically set by range re-segmentation. [↑](#footnote-ref-8)
10. The IBSI has not introduced image transformation into the standardised image processing scheme, and is in the process of benchmarking various common filters. This section may therefore be expanded in the future. [↑](#footnote-ref-9)
11. A software is compliant if and only if it is able to reproduce the feature reference values for the digital phantom and for one or more image processing configurations using the radiomics CT phantom. Reviewers may demand that you provide the IBSI compliance spreadsheet for your software. [↑](#footnote-ref-10)
